# CNS axon regeneration inhibitors stimulate an immediate early gene response via MAP kinase-SRF signaling

**DOI:** 10.1186/s13041-014-0086-6

**Published:** 2014-11-19

**Authors:** Sina Stern, Bernd Knöll

**Affiliations:** Department Molecular Biology, Eberhard-Karls-University Tübingen, Interfaculty Institute for Cell Biology, Auf der Morgenstelle 15, 72076 Tübingen, Germany; Current address: Ulm University, Institute for Physiological Chemistry, 89081 Ulm, Germany; Current address: German Centre for Neurodegenerative Diseases (DZNE), Ludwig-Erhard-Allee 2, 53175 Bonn, Germany

**Keywords:** SRF, Immediate early gene, Axon, Regeneration, MAP kinase, Neuron, Myelin, c-Fos

## Abstract

**Background:**

CNS axon regeneration inhibitors such as Nogo and CSPGs (Chondroitin Sulfate Proteoglycans) are major extrinsic factors limiting outgrowth of severed nerve fibers. However, knowledge on intracellular signaling cascades and gene expression programs activated by these inhibitors in neurons is sparse. Herein we studied intracellular signaling cascades activated by total myelin, Nogo and CSPGs in primary mouse CNS neurons.

**Results:**

Total myelin, Nogo and CSPGs stimulated gene expression activity of the serum response factor (SRF), a central gene regulator of immediate early (IEG) and actin cytoskeletal gene transcription. As demonstrated by pharmacological interference, SRF-mediated IEG activation by myelin, Nogo or CSPGs depended on MAP kinase, to a lesser extent on Rho-GTPase but not on PKA signaling. Stimulation of neurons with all three axon growth inhibitors activated the MAP kinase ERK. In addition to ERK activation, myelin activated the IEG c-Fos, an important checkpoint of neuronal survival vs. apoptosis. Employing *Srf* deficient neurons revealed that myelin-induced IEG activation requires SRF. This suggests an SRF function in mediating neuronal signaling evoked by axon regeneration associated inhibitors. Besides being a signaling target of axon growth inhibitors, we show that constitutively-active SRF-VP16 can be employed to circumvent neurite growth inhibition imposed by myelin, Nogo and CSPGs.

**Conclusion:**

In sum, our data demonstrate that axon regeneration inhibitors such as Nogo trigger gene expression programs including an SRF-dependent IEG response via MAP kinases and Rho-GTPases.

## Background

Upon CNS injury, myelin- and astrocyte-associated inhibitors limit re-growth of lesioned nerve fibers [1]. Myelin-associated inhibitors such as Nogo signal through the Nogo receptor complex (NgR) or PIR-B (paired immunoglobulin-like receptor B) expressed on CNS neurons [2]. Astrocyte-derived CSPGs activate the transmembrane protein tyrosine phosphatase receptor, RPTPσ, as well as NgRs to prevent axon growth [2]. Once activated, the intracellular signaling pathways employed by these receptors are not well understood. So far, Rho-GTPase and Rho kinase (ROCK) as well as PKC signaling are known to be recruited by NgR receptors [3]. Further downstream LIM kinase-cofilin signaling is connecting NgR with the actin cytoskeleton thereby contributing to axon stalling [4].

Notably many other signaling pathways including cAMP/PKA, PTEN/AKT, mTOR, GSK3β and MAP kinase signaling have been implicated in axon regeneration [3]. For instance, elevating neuronal cAMP levels emerges as potent mechanism to bypass axon injury [5]. In addition, MAP kinases such as p38, ERK and JNK are involved in CNS axon regeneration [6-8]. ERK is involved in peripheral [9-11] and central [12-14] axon regeneration. However it has not been investigated in much detail whether these signaling cascades are activated upon NgR, RPTPσ or PIR-B engagement by their ligands. In this study we analyzed whether Rho-GTPase, cAMP/PKA and MAP kinase signaling are mediating signaling upon stimulation of primary CNS neurons with total CNS myelin, purified Nogo and CSPGs.

In addition, we investigated whether these axon regeneration inhibitors modulate gene expression. So far, activation of NgRs, PIR-B or RPTPs has not been connected to modulation of gene expression. We focused on SRF (serum response factor), a gene regulator mediating an immediate early gene (IEG) response of e.g. c-Fos [15], a hallmark of neuronal activation as well as regulatory switch of neuronal apoptosis vs. survival [16]. SRF cooperates with TCFs (ternary complex factors) such as Elk-1 to convey IEG induction. Besides IEGs, SRF regulates actin cytoskeletal gene abundance [17]. SRF has not been studied in CNS axonal regeneration so far. In PNS axon regeneration, using facial nerve regeneration as model system, we demonstrated a stimulatory SRF function in motoneuron survival [18] and axonal regeneration [19] involving a cytoplasmic SRF localization [19].

Here we show that stimulation of CNS neurons with total myelin, Nogo or CSPGs activated SRF-dependent *c-Fos* reportergene activity. Pharmacological inhibition of MAP kinase, and to la lesser extent Rho-GTPase/ROCK, but not cAMP/PKA signaling prevented SRF gene activity induced by all three inhibitors. MAP kinases (i.e. ERK) were activated upon incubation of neurons with myelin, Nogo or CSPGs. Further downstream of ERK we observed c-Fos induction by myelin, a process blocked by SRF ablation. Finally, we show that SRF is not only a signaling target of axon regeneration inhibitors. Employing constitutively-active SRF-VP16 circumvented neurite growth impaired by myelin, Nogo and CSPGs. This provides first *in vitro* data unraveling an SRF potential in CNS axon regeneration.

## Results

### Axon regeneration inhibitors activate SRF-dependent gene activity

**Figure 1:**
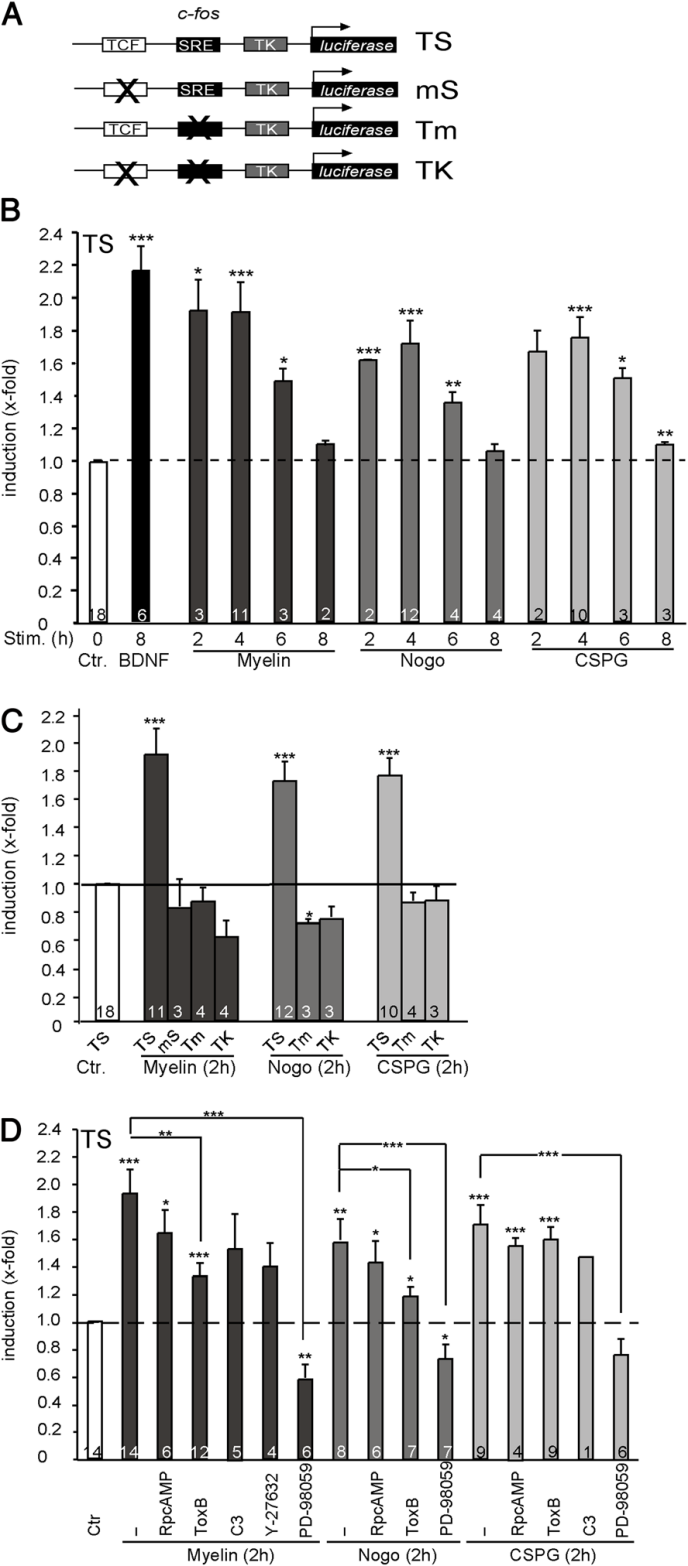
**Axon regeneration inhibitors enhance SRF mediated gene activity. (A)** Reportergene assays were performed using a *c-Fos* derived construct containing a TCF and SRF (“TS”), TCF (“Tm”) or SRF binding site (“mS”). **(B)** Myelin, Nogo and CSPGs enhance TCF-SRF gene activity as revealed by the “TS” reportergene construct. **(C)** Mutating the TCF or SRF binding sites abolished induction upon a 2 h stimulation with either axon regeneration inhibitor. **(D)** The signaling cascade underlying myelin, Nogo and CSPG mediated TCF-SRF promoter activity involves MAP kinases and to some extent Rho-GTPases. MAP kinase signaling was blocked by PD-98059. Rho-GTPase signaling was inhibited via application of ToxB (all Rho-GTPases), C3 (RhoA only), or Y-27632 (targeting ROCK). PKA signaling, interfered with by Rp-cAMPS incubation, was dispensable for signaling to SRF. Numbers in bars indicate independent cell cultures analyzed.

We employed SRF dependent reportergene assays to study whether total myelin, Nogo or CSPG modulate SRF activity in primary cerebellar neurons (Figure 1). For this, the *c-fos* promoter was connected to a luciferase-based reportergene construct (Figure 1A). The *c-fos* promoter (“TS”) harbors a TCF binding site (“T”) and a serum response element, SRE (“S”; Figure 1A). Neurons were stimulated for various time-points (2-8 h) with these regeneration inhibitors or with the known SRF stimulus BDNF [20,21].

All three axon regeneration inhibitors enhanced SRF gene activity of the *c-fos* reportergene containing both SRF and TCF binding sites (“TS”) with shortest stimulation periods (2 and 4 h) being most effective (Figure 1B). Of note, all three inhibitors activated SRF to a comparable level as achieved with BDNF (Figure 1B). Overall SRF expression levels or nuclear SRF localization was not affected by inhibitor (i.e. CSPG) application (data not shown).

Next, we assessed whether SRF alone is sufficient to activate the *c-fos* promoter or whether SRF requires TCF cofactors (Figure 1C). For this, constructs lacking either the TCF (mS; “m” for mutated) or SRF (Tm) binding site were employed (see Figure 1A). Mutation of either the TCF or the SRF binding site abolished reportergene activity upon 2 h stimulation (Figure 1C). This suggests that myelin, Nogo or CSPG induced gene activity requires SRF-TCF interaction.

In order to single out pathways connecting myelin, Nogo or CSPG signaling with SRF gene regulation, pharmacological interference was employed (Figure 1D). Interference with cAMP/PKA signaling was achieved by pre-incubating neurons with Rp-cAMPS. We applied ToxB to block all three major Rho-GTPases (RhoA, Rac and Cdc42) or C3 to specifically target RhoA only. In addition, the RhoA effector ROCK was inhibited via Y-27632 bath application. Inhibition of MAP kinase signaling was accomplished by PD-98059, affecting MEK activation. Results obtained demonstrate a similar dependence of all three inhibitors on the inspected signaling pathways (Figure 1D). In the presence of Rp-cAMPS, all three inhibitors still induced SRF reportergene activity indicating that cAMP/PKA signaling is dispensable for SRF activation (Figure 1D). In contrast, interfering with Rho-GTPase signaling reduced, although not completely prevented, all three inhibitors from activating SRF (Figure 1D). Interestingly, abolishing MAP kinase signal propagation completely prevented all three inhibitors from stimulating SRF (Figure 1D). This suggests that upon activation by myelin, Nogo or CSPG, receptors recruit the MAP kinase pathway to connect surface activation with nuclear SRF signaling.

### Myelin, Nogo or CSPG activate MAP kinases in primary CNS neurons

**Figure 2:**
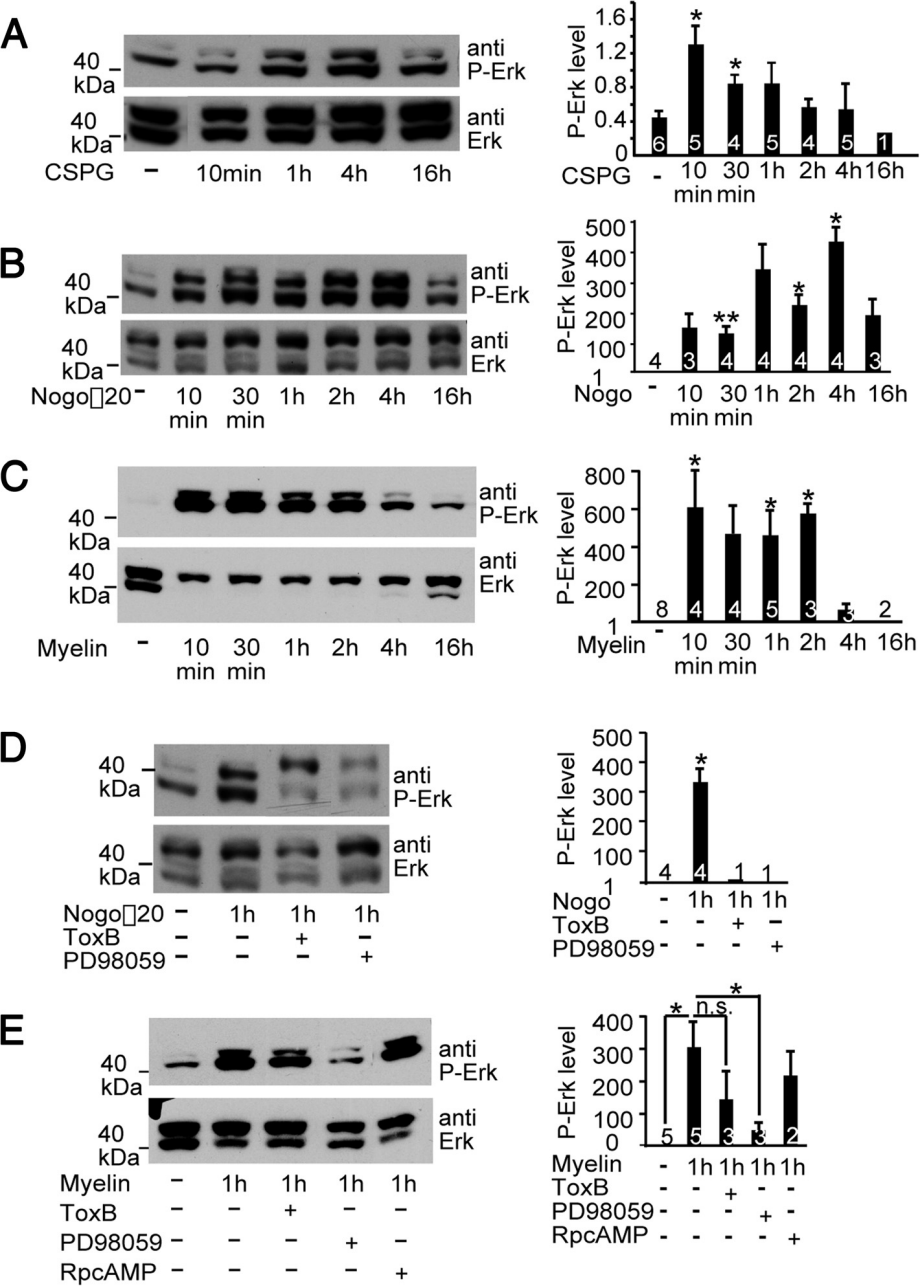
**Axon regeneration inhibitors enhance ERK activity. (A-C)** Cerebellar neurons were incubated with CSPGs **(A)**, Nogo **(B)** or myelin **(C)** for indicated time-points followed by Western Blotting for total and activated ERK (P-ERK). All three axon regeneration inhibitors activated ERK with shorter incubation periods being more effective. Bar diagrams depict P-ERK levels normalized to total ERK levels for individual experiments. **(D,E)** Cerebellar neurons were stimulated with Nogo **(D)** or myelin **(E)** for 1 h. Cultures were pre-incubated with pharmacological inhibitors for 15 mins. Interference with Rho-GTPase (ToxB) and more pronounced MAP kinase (PD98059) signaling, but not cAMP/PKA signaling (Rp-cAMPS), blocked Nogo **(D)** or myelin’s **(E)** potential to fully activate ERK. Numbers in bars indicate independent cell cultures analyzed.

Data obtained above suggest that axon regeneration inhibitors recruit MAP kinases to connect receptor activation at the neuronal surface with SRF gene regulation. To corroborate this, we assessed whether application of these inhibitors to primary neurons results in activation of the MAP kinase ERK (Figure 2). For this, cerebellar neurons were stimulated with myelin, Nogo or CSPGs followed by Western Blotting for activated, i.e. phosphorylated ERK (P-ERK).

Indeed, all three inhibitors up-regulated P-ERK with earlier stimulation time-points (10 mins - 2 h) being more effective than later time-points (4-16 h; Figure 2A-C). This suggests that upon activation of their cognate receptors myelin, Nogo and CSPGs transiently activate a MAP kinase signaling cascade in neurons.

MAP kinases and Rho-GTPase signaling is interconnected at multiple levels. Hence, we investigated a potential crosstalk of Rho-GTPases and MAP kinases upon axon regeneration inhibitor signaling (Figure 2D,E). Expectedly, MEK inhibition (via PD98059) reduced P-ERK levels (Figure 2D,E). In addition, blockage of all three major Rho-GTPases via ToxB but not cAMP/PKA signaling also reduced Nogo (Figure 2D) and myelin (Figure 2E) induced ERK activation. This suggests that MAP kinase signaling induced by axon regeneration inhibitors requires Rho-GTPases to achieve full activation.

### Myelin evokes an SRF dependent IEG response

**Figure 3:**
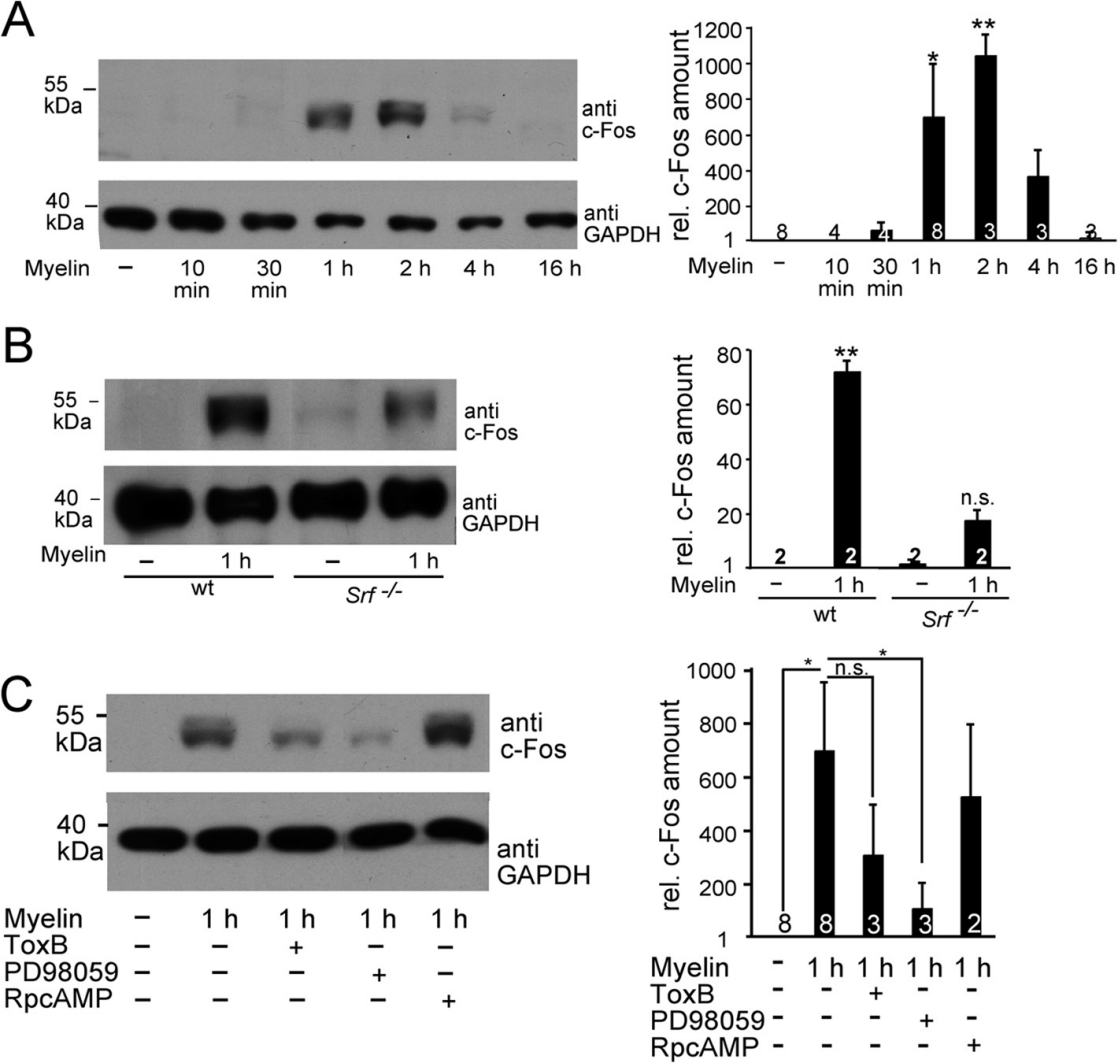
**Myelin induces an IEG response of c-Fos involving SRF.** Cerebellar neurons were stimulated with total myelin for the indicated time-points followed by analysis of c-Fos protein abundance. **(A)** Myelin induces c-Fos after 1 and 2 h of application. At later time-points, c-Fos is down-regulated as also depicted in the quantification of an individual experiment. **(B)** In *Srf* deficient neurons, myelin fails to induce c-Fos to a similar extent as observed in wild-type neurons. **(C)** Myelin recruits MAP kinases and Rho-GTPase but not cAMP/PKA signaling to induce c-Fos. Numbers in bars indicate independent cell cultures analyzed.

Activated ERK kinase stimulates gene regulators thereby inducing a first gene expression wave. Rapid but transient IEG induction is such a first molecular response initiated in neurons upon e.g. injury [16]. We asked whether confrontation of neurons with myelin also stimulates a neuronal IEG response. For this, cerebellar neurons were incubated for various time-points (10 mins - 16 h) with myelin followed by quantification of c-Fos protein abundance (Figure 3). C-Fos protein is absent in unstimulated neurons (Figure 3A). However, myelin strongly induced c-Fos at 1 and 2 h of application, whereas at later time-points c-Fos was not detectable (Figure 3A).

C-Fos is an SRF target gene (see Background), thus myelin might engage SRF to induce c-Fos. Indeed, c-Fos induction is reduced in *Srf* mutant neurons (Figure 3B). As c-Fos induction is not completely abolished, other gene regulators in addition to SRF might contribute to myelin-evoked IEG induction. To decipher upstream signaling events involved in myelin-induced IEG induction we interfered with Rho-GTPase (Tox B) and MAP kinase (PD98059) signaling (Figure 3C). Pre-incubation with either signaling inhibitor abolished myelin-induced c-Fos induction suggesting that both MAP kinase and Rho-GTPase signaling are required for myelin signaling (Figure 3C). In contrast cAMP/PKA signaling is dispensable (Figure 3C) in line with our previous results (Figures 1 and 2).

In summary, myelin triggers a neuronal IEG response mediated via Rho-GTPase, MAP kinase and SRF signaling.

### SRF-VP16 expression overcomes neurite growth inhibition exerted by myelin, Nogo and CSPGs

Results obtained so far (Figures 1, 2 and 3) suggest that a first response of CNS neurons upon encounter of axon regeneration inhibitors is a rapid but transient IEG induction. SRF also governs transcriptional responses allowing prolonged cellular adaptions upon neuronal stimulation. The latter involves SRF’s access towards modulating actin dynamics via directly controlling mRNA abundance of genes encoding actin isoforms or actin binding proteins [15,17]. SRF participates in various actin-based motility processes including cell migration, neurite growth, growth cone motility and axon guidance [15]. Thus we wondered whether a constitutively-active SRF protein variant, not subject to endogenous regulatory mechanisms, might alleviate impaired neuronal motility evoked my myelin, Nogo and CSPGs. We employed SRF-VP16, a fusion protein consisting of SRF and the viral VP16 transactivation domain. Off-target effects caused by VP16 were controlled by SRF-VP16-ΔMADS harboring the VP16 domain, yet lacking DNA binding activity [22,23].

**Figure 4:**
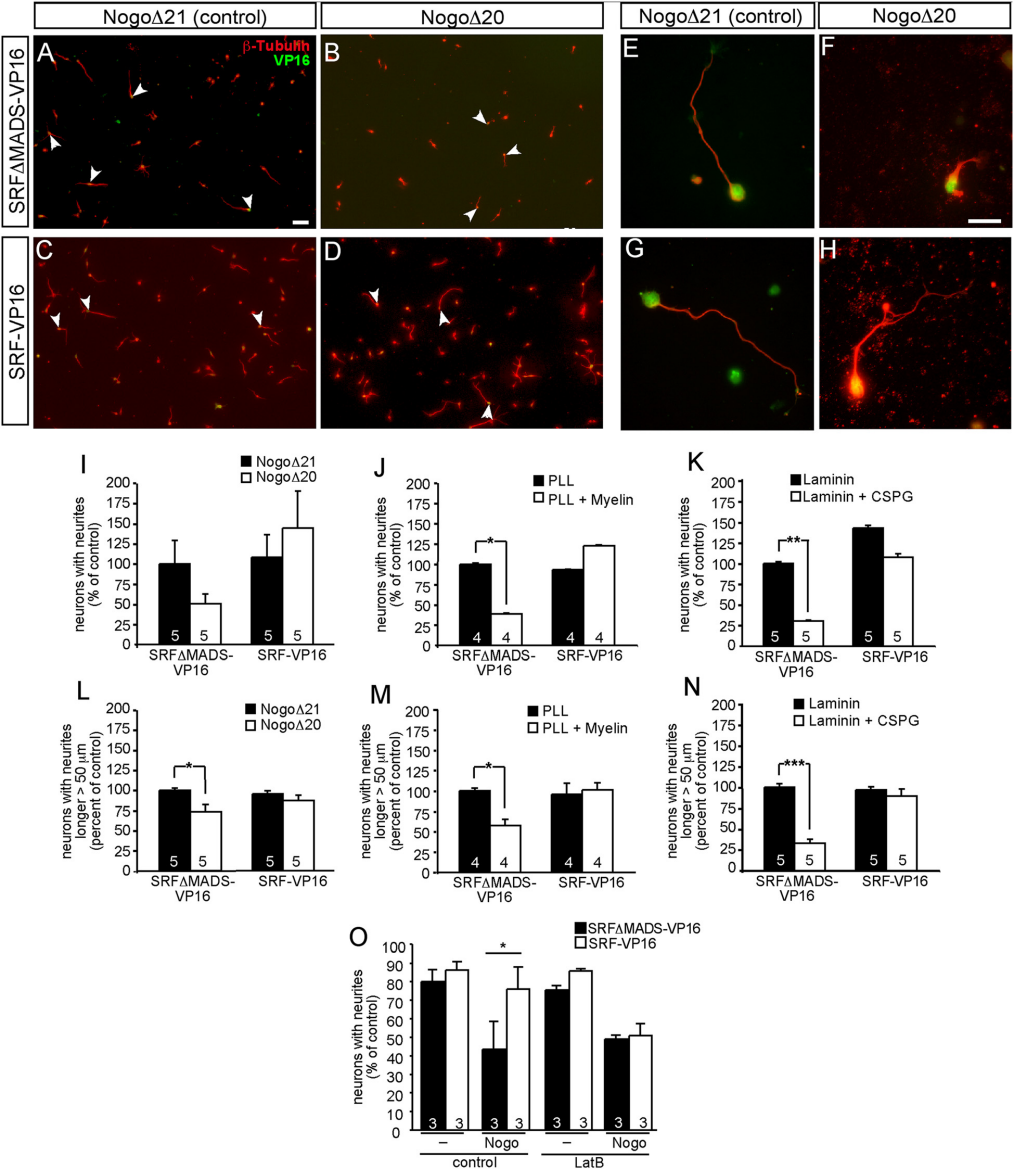
**SRF-VP16 overcomes myelin- and astrocyte-associated neurite growth inhibition** ***in vitro*** **.** Primary neurons were plated on Nogo, total myelin and CSPGs, followed by staining for expression of SRF via the VP16 domain and tubulin to visualize the entire neuron. **(A-H)** Primary cerebellar neurons were grown on either an inactive Nogo peptide (NogoΔ21; **A**, **C**, **E**, **G**) or the growth-inhibiting peptide NogoΔ20 **(B, D, F, H)**. Neurons were expressing either the control protein SRFΔMADS-VP16 **(A, B, E, F)** or SRF-VP16 **(C, D, G, H)**. Neurons were stained for SRF (green) and tubulin (red). The active Nogo peptide **(B, F)** reduced neurite growth of SRF-ΔMADS-VP16 expressing neurons compared to the permissive control substrate NogoΔ21 **(A, E)**. Expression of SRF-VP16 stimulated neurite growth on the inhibitory Nogo substrate **(D, H)**. Arrowheads point at individual VP16-positive neurons. **(E-H)** represent higher magnification images of individual neurons with nuclear SRF localization (in green). **(I-N)** Quantification of neurite growth on Nogo **(I, L)**, myelin **(J, M)** and CSPGs **(K, N)**. In **(I, J, K)** percentage of neurons with neurite growth is plotted for the various conditions. In **(L, M, N)** neurite length was quantified by taking only neurons into account with neurites grown longer than 50 μm. In each bar diagram, the condition reflecting control substrate and SRFΔMADS-VP16 was set to 100%. **(O)** SRF-VP16 requires actin dynamics to overcome Nogo-mediated neurite growth inhibition. In the presence of the actin depolymerizing agent Latrunculin B (Lat B), SRF-VP16 failed to elevate neurite outgrowth inhibited by Nogo. Numbers in bars depict independent cultures analyzed for each condition. Standard error is provided. Scale-bar **(A-D)** =100 μm; **(E-H)** =50 μm.

We employed *in vitro* assays to test whether SRF can augment neurite growth in a growth-inhibitory environment mimicking the inhibitory environment encountered by lesioned axons *in vivo* (Figure 4). Here, primary neurons were plated on coverslips coated with total myelin, Nogo or CSPG. Neurons expressing the control protein, SRF-VP16-ΔMADS (arrowheads in Figure 4A; higher magnification Figure 4E) grew on the Nogo control peptide NogoΔ21 [24]. In contrast, an active fragment derived from the Nogo protein (NogoΔ20) decreased neurite outgrowth of cerebellar (Figure 4B,F) but not hippocampal neurons (data not shown). SRF-VP16 expression overcame this neurite growth inhibition by NogoΔ20 (Figure 4D,H). SRF-VP16 expressing neurons on the inhibitory Nogo substrate (arrowheads in Figure 4D,H) almost achieve neurite growth comparable to the growth permissive control substrate (Figure 4A,E). In addition to Nogo (Figure 4A-L), we employed total myelin (Figure 4J,M) and CSPGs (Figure 4K,N). Similar to Nogo, SRF-VP16 alleviated growth inhibition on neurites exerted by myelin and CSPGs (Figure 4J-N). Cell survival measured by quantification of active-caspase 3 protein levels revealed no difference between SRF-VP16-ΔMADS and SRF-VP16 expressing cells (data not shown).

SRF and actin dynamics are intertwined (see Background). Hence, we asked whether SRF-VP16 requires actin treadmilling for overcoming neurite outgrowth inhibition *in vitro* (Figure 4O). Indeed, interfering with actin polymerization by LatrunculinB application abolished SRF-VP16’s capacity to stimulate neurite growth in a regeneration-inhibitory environment presented by Nogo (Figure 4O). This suggests that SRF-VP16 modulates the actin cytoskeleton to enhance axonal regeneration *in vitro*.

These experiments revealed that SRF-VP16 enhances neurite growth blocked by axon regeneration inhibitors. Thus SRF can alleviate the impact of extrinsic cues blocking axon regeneration (Figure 4) as well as modulate neuron’s intrinsic growth potential as shown before [22,25,26].

## Discussion

In recent years many extrinsic factors posing regeneration obstacles to injured CNS axons were identified [27]. However how these myelin- or astrocyte-associated molecules transmit their axon growth prohibiting activity within neurons is not understood in much molecular depth. So far Rho-GTPases have been the main signaling intermediate identified in neurons to connect these extrinsic factors with intrinsic signaling programs resulting in axonal stalling [3].

Here, we employed CNS neurons to analyze which signaling pathways are activated when neurons encounter the regeneration inhibitors total myelin, Nogo or CSPGs. We further investigated whether these regeneration inhibitors modulate gene expression, an event largely neglected in CNS compared to PNS axon regeneration. Our data show that all three regeneration obstacles stimulated SRF-dependent gene expression (Figure 1). Interestingly, both, growth-promoting BDNF as well as growth-inhibiting regeneration inhibitors stimulated SRF, yet with different temporal patterns (Figure 1A). Axonal growth inhibitors activated SRF only at shortest but failed to activate SRF at longer time-points (8 h; Figure 1A). In contrast, BDNF typically provides sustained SRF activation ranging from short time-points [28] to long-term stimulation (Figure 1A). Such prolonged BDNF-mediated SRF activity and thereby overall cellular response might eventually enhance neuronal survival. In opposite to this, axonal growth inhibitors stimulate a rapid but transient IEG response (Figure 1A). As IEGs are well-known molecules regulating a cellular switch between cell death and survival [16], axonal growth inhibitors might recruit IEGs to modulate an initial cellular response mediating axonal regeneration. Notably, both BDNF and axonal growth inhibitors employ small Rho family GTPases as downstream signaling effectors [1,29]. Rho-GTPases are known to activate F-actin polymerization which in turn will enhance SRF activity [17,30]. Thus, despite different time-scale of operation, pro- and anti-growth signals might share Rho-GTPase-to-SRF signaling as common downstream effector.

Axonal growth inhibitors did not modulate SRF activity through alterations in either SRF’s nuclear abundance nor nuclear localization (data not shown). Instead, our data are congruent with models of SRF activation involving specific cofactor recruitment to stimulate SRF-dependent gene transcription [15,17]. Using reportergene assays (Figure 1C), we demonstrate an interaction of SRF with TCF family transcription factors to convey an axon regeneration inhibitor mediated IEG response. TCF family members such as Elk-1 are activated through MAP kinase phosphorylation [15,17]. In this study, MAP kinases were identified as critical downstream effectors activated by myelin, Nogo and CSPGs (Figures 1, 2 and 3). Thus, besides Rho-GTPases, MAP kinases emerge as a further signaling pathway activated by myelin- and astrocyte associated growth inhibitors. Our data suggest that such MAP kinase activation might eventually result in TCF phosphorylation and TCF-SRF mediated gene transcription.

In this study, we have not addressed which of the receptors (i.e. NgRs, PIR-B or RPTPs) are engaged by axon regeneration inhibitors. However, we noted that oligodendrocyte- (total myelin and Nogo) as well as astrocyte-derived (i.e. CSPGs) axon growth inhibitors all shared a similar signaling profile with regard to intermediates recruited (i.e. MAP kinase, Rho-GTPase, SRF) and temporal signaling sequence followed (i.e. shorter stimulation time-points were more effective than pro-longed stimulation). This result suggests that all regeneration inhibitors signal through the same receptor molecules, a finding supporting the current model that all regeneration inhibitor indeed share the same neuronal receptor [2].

How do these *in vitro* findings relate to axon injury *in vivo*?

Induction of an IEG response is reported in various brain injuries including spinal cord injury [31]. Rapid but transient induction of c-Fos is a key event in regulation of survival vs. elimination of injured neurons [16]. Thus, our *in vitro* results suggest that upon encounter of myelin- or astrocyte-associated regeneration inhibitors, propagation of such an IEG response in neurons might also require MAP kinases and SRF *in vivo* (Figure 3). In such a scenario, SRF might be an effector fulfilling the detrimental impact of signaling initiated by axon growth inhibitors. However, our data also reveal that SRF might be employed to circumvent the growth inhibitory potential of these regeneration obstacles. For this constitutively-active SRF-VP16 was used which unlike wild-type SRF is not subject to neuron-endogenous regulatory mechanisms. SRF-VP16 rescued myelin, Nogo or CSPG evoked neurite growth inhibition in primary neurons (Figure 4). The latter is likely due to SRF’s potential to modulate neuronal actin cytoskeletal dynamics [17,22]. For instance, SRF-VP16 enhances the cellular F-actin content [23] and the activity of the actin severing factor cofilin [32] and might thereby allow for neurite growth on inhibitory substrates. In line with a beneficial SRF role in axonal regeneration, we demonstrated before an enhanced motoneuron survival by SRF-VP16 and impaired axon regeneration in *Srf* deficient mice in the peripheral nervous system [18,19].

In sum our data show that extrinsic regeneration barriers activate a neuronal signaling pathway involving MAP kinases and Rho-GTPases. In addition we show for the first time that axon growth inhibitors such as Nogo elicit a neuronal gene expression program mediated by SRF. Due to its dual access to regulation of IEGs and actin cytoskeletal dynamics, SRF might be an interesting gene regulator to analyze in CNS axon regeneration *in vivo*.

## Methods

### Cell biology

P3-P6 cerebellar or P1-P3 wild-type or *Srf* mutant hippocampal neurons were used. Reportergene assays were performed as described in [33,34]. Before plating, cerebellar neurons were electroporated with 12.8 μg luciferase construct (kind gifts of A. Nordheim, Tübingen University, Germany) and 3.2 μg β galactosidase. Luciferase values were measured 48 h later and normalized to the β galactosidase control. Cells were stimulated with 10 ng/ml BDNF (Peprotech, Hamburg, Germany), 12 μg/ml myelin [35], 10 μg/ml CSPGs (Millipore) or 63 μg/ml Nogo. The active peptide Nogo∆20, and the control peptide Nogo∆21, were kindly provided by Dr. M. Schwab (ETH Zurich, Switzerland) and purified as described before [24]. Cells were pre-incubated for 15 minutes with PD-98059 (100 μM, Enzo Life Sciences, Lörrach, Germany), Rp-cAMPS (50 μM, Sigma), ToxB (50 ng/ml, Sigma), C3-Transferase (2 μg/ml, Cytoskeleton) or Y-27632 (16 mM, Sigma).

For neurite growth assays on myelin and CSPGs, hippocampal, whereas cerebellar neurons were used for Nogo experiments. Neurons were grown on PLL-coated (100 μg/ml) coverslips followed by coating with inhibitory substances as follows. 4 μg myelin was dried on coverslips over night using an exicator. Both Nogo peptides were used at 35 μg/ml in HBSS for 1 h at 37°C. After washing with HBSS, coverslips were coated with 5 μg/ml laminin. The CSPGs were applied at 5 μg/ml in PBS overnight at 4°C followed by laminin coating as above. Before plating, neurons were electroporated using Amaxa mouse neuron nucleofector solution as recommended by the manufacturer (Amaxa, Cologne, Germany). 3 μg of the indicated plasmids were electroporated. After 24 h to 48 h in the incubator, immuncytochemistry was performed. Latrunculin B (Sigma) was applied at 0.2 μM for 16 h in the culture medium.

### Biochemistry

Protein lysates were prepared as described before [33]. Rabbit anti-active ERK (Cell Signaling; 1:1000), rabbit anti-ERK (Cell Signaling; 1:1000), rabbit anti c-Fos (Calbiochem; 1:5000) and mouse anti-GAPDH (Acris; 1:50.000) antibodies were used.

### Statistics

Numbers of independent experiments or animals are indicated in the results section and figure bars. For cell culture experiments, at least three independent experiments were performed. In each experiment at least 30 neurons were analyzed. Statistical significance was calculated using two tailed t test with *, **, *** indicating p ≤0.05, 0.01 and 0.001, respectively. Standard deviation (Figures 1 and 4) or standard error (Figures 2 and 3) is provided if not mentioned otherwise.
